# The enhancive effect of the 2014–2016 El Niño-induced drought on the control of soil-transmitted helminthiases without anthelmintics: A longitudinal study

**DOI:** 10.1371/journal.pntd.0012331

**Published:** 2024-07-12

**Authors:** Attarat Pattanawongsa, Pattanasak Kammaneechan, Prasit Na-ek, Blego Sedionoto, Witthaya Anamnart

**Affiliations:** 1 School of Pharmacy, Walailak University, Nakhon Si Thammarat, Thailand; 2 Drug and Cosmetics Excellence Center, Walailak University, Nakhon Si Thammarat, Thailand; 3 School of Public Health, Walailak University, Nakhon Si Thammarat, Thailand; 4 Department of Medical Science, School of Medicine, Walailak University, Nakhon Si Thammarat, Thailand; 5 Research Center in Tropical Pathobiology, Walailak University, Nakhon Si Thammarat, Thailand; 6 Department of Environmental Health, Faculty of Public Health, Mulawarman University, Samarinda, East Kalimantan, Indonesia; 7 School of Allied Health Sciences, Walailak University, Nakhon Si Thammarat, Thailand; Huazhong University of Science and Technology Tongji Medical College, CHINA

## Abstract

**Background:**

Soil-transmitted helminthiases (STHs) are common in tropical and subtropical regions. Southern Thailand experiences an extended rainy season, leading to persistently moist soil. This condition supports the life cycle of STHs, hindering effective control due to reinfection and low drug efficacy. We implemented a novel STH control strategy during the dry season aimed at decreasing reinfection rates without enhancing sanitation or hygiene practices. However, there were unexpected, prolonged droughts linked to El Niño events from 2014 to 2016. Additionally, we assessed the effects of these drought conditions on further control measures without the use of anthelmintics.

**Methodology/Principal findings:**

A longitudinal study was conducted from 2012 to 2016. Stool samples collected from 299 participants were analyzed using the Kato-Katz and agar plate culture methods. Participants who tested positive for STHs received a single 400 mg dose of albendazole. The efficacy of the treatment was evaluated three weeks later. To confirm the control measures were implemented during the dry season, we monitored the number of rainy days following albendazole treatment for 52 days, of which 38 were without rain. Follow-up stool examinations were carried out in 2013 and 2016, with no additional doses of albendazole administered. Rainfall and rainy day data, which served as indicators of unexpected droughts due to El Niño, were collected from the nearest local meteorological stations. Before the drought, there was a decrease in STH prevalence in 2013—except for trichuriasis—attributable to the dry season control efforts. Despite these efforts, STH prevalence remained high. Remarkably, in 2016, following the drought period, the prevalence of trichuriasis, which had not changed previously, spontaneously declined without further albendazole treatment compared to 2013.

Furthermore, the prevalence of strongyloidiasis remained unchanged likely due to its low susceptibility to drought conditions, as it can reproduce within hosts. Conversely, the prevalence of other STHs consistently declined. The drought and possible improvements in sanitation and hygiene practices contributed to this decrease by reducing rates of reinfection and new infection and by increasing the natural cure rate. Additionally, some participants infected with hookworms or *Trichuris* who were not cured by albendazole experienced natural remission.

**Conclusions/Significance:**

Control measures implemented during the dry season, combined with a 14-month-long drought induced by the El Niño event of 2014–2016, and some improvements in sanitation and hygiene practices, contributed to a decrease in both the prevalence and intensity of STHs, except for *S*. *stercoralis*. Over time, *S*. *stercoralis* is likely to become the predominant species among the STHs.

## Author summary

Soil-transmitted helminths (STHs) are prevalent in tropical rainforest regions of southern Thailand, where frequent rainfall can enhance the environmental life cycle of STHs, leading to reinfection. To mitigate this, we implemented control measures during the dry season from 2012 to 2016, aiming to reduce reinfection rates. For STH detection, we used agar plate culture and the Kato-Katz methods to analyze stool samples collected from 299 participants. Only those who tested positive for STHs received a single dose of albendazole, without accompanying health education, sanitation, or hygiene interventions. The effectiveness of the treatment was evaluated three weeks later. Follow-up assessments were conducted in 2013 and 2016 without further administration of albendazole. In 2013, a year after the albendazole treatment, there was a decrease in the prevalence of all STHs, except for trichuriasis. Unexpectedly, the El Niño event from 2014–2016 triggered prolonged droughts from 2014 to 2015, which likely led to a reduction in the prevalence and intensity of all STHs, excluding *S*. *stercoralis*. This exception was due to its low natural cure rate (NCR), as it can reproduce within the host. Additionally, the drought likely led to a reduction in the reinfection rate and an increase in the NCR. Notably, the NCR of trichuriasis induced by drought was more significant than that achieved through albendazole treatment. Furthermore, participants infected with hookworm or *T*. *trichiura* who were not cured by albendazole experienced natural remission. Improvements in sanitation and hygiene also likely contributed to the decreased prevalence of STHs. Based on these findings, we recommend that the control of STHs, especially in tropical rainforests characterized by long rainy seasons and persistently wet soil, should be strategically timed during the dry season to optimize effectiveness.

## Introduction

Soil-transmitted helminths (STHs) comprise species such as *Ascaris lumbricoides*, *Trichuris trichiura*, hookworm, and *Strongyloides stercoralis*. In 2010, global infections were estimated at approximately 819 million for *A*. *lumbricoides*, 464.4 million for *T*. *trichiura*, and 438.9 million for hookworm. Additionally, out of 4.98 million years lived with disability (YLDs) attributed to STHs, 65%, 22%, and 13% were caused by hookworms, *A*. *lumbricoides*, and *T*. *trichiura*, respectively [1]. It is estimated that 30–100 million people globally are infected with *S*. *stercoralis* [2]. The hookworm species *Necator americanus* and *Ancylostoma duodenale* are particularly noteworthy, with *N*. *americanus* being prevalent in Asia, the sub-Saharan region of Africa, and Central and South America, and is the dominant species in our study areas in southern Thailand [3,4]. STHs are most common in tropical and subtropical regions, especially in low-income and rural areas. The transmission of STHs is influenced by several factors including poor sanitation and hygiene, inadequate water supply, urbanization, and climatic and environmental conditions [5,6]. Climatic factors are crucial for the control of STHs as the development and survival of all larval stages require moisture, which is typically maintained by rainfall in the environment where the stool containing these larvae is deposited on wet soil [6,7]. The climate in Nakhon Si Thammarat (NST) on the eastern coast of southern Thailand is classified as tropical rainforest, while other provinces feature tropical monsoon climates. The various parts of Thailand outside these regions generally experience tropical savanna climates [8]. Consequently, NST receives significantly more rainfall than other provinces on the eastern coast of southern Thailand, which exceeds the average rainfall for the country (S1 Table). This heavy rainfall contributes to a 10-month rainy season in the tropical rainforest region of NST, resulting in persistently wet soil throughout most of the year. In contrast, the tropical savanna climate found in most parts of Thailand outside the southern region experiences a shorter, five-month rainy season, followed by a dry season lasting at least seven months, resulting in predominantly dry soil. The prolonged periods of wet soil in NST compared to shorter periods in other regions are likely a critical factor in the 3–10 times higher prevalence of STHs in the southern region than in other parts of Thailand [9,10]. This higher prevalence is likely exacerbated by increased rates of reinfection and reduced efficacy of albendazole (ABZ), which poses significant challenges to our STH control efforts.

The control of STHs includes preventive chemotherapy (PC), public health education, and interventions in water, sanitation, and hygiene (WASH) [11]. The World Health Organization recommends PC as a primary strategy [12], although reinfection often occurs within months to years following treatment [13]. Consequently, combining PC with WASH interventions is advised for more effective control of STHs [11]. In our study area, all individuals over the age of two received PCs from public health officials in 2006–2007 in accordance with the policy of the Ministry of Public Health. This involved administering a single dose of 400 mg albendazole every six months. Despite these efforts, when we conducted parasitological diagnostics for human strongyloidiasis from 2008 to 2011, the prevalence of STHs remained high. This persistent prevalence can likely be attributed to inadequate sanitation, poor hygiene practices, and impoverished living conditions. The participant information sheet indicated that those testing positive for any helminth eggs would receive anthelmintic treatment. Specifically, albendazole was administered to positive participants three times over three years, corresponding to the long rainy season and the generally wet soil conditions prevalent throughout the study region. We focused on hookworm infections, monitoring both the treatment outcomes and rates of reinfection. Our findings showed that 13–18% of treated participants were not cured by the treatment, and 80–90% of those initially cured experienced reinfection within six months. While reinfected participants were consistently cured with each round of albendazole, those who remained uncured after initial treatments continued to be uncured despite receiving two to three rounds of albendazole. Notably, uncured participants typically exhibited low mean corpuscular volume (MCV) and mean corpuscular hemoglobin (MCH), suggesting they might be carriers of abnormal hemoglobin, whereas cured cases usually had normal MCV and MCH levels. These uncured participants serve as reservoirs for ongoing hookworm transmission. This raises further questions about the impact of climatic factors, such as frequent rainfall in this tropical rainforest area, which may have contributed to the ineffectiveness of previous STH control efforts in 2006–2007. Additionally, we gathered feedback regarding anthelmintic intake. Our findings revealed several challenges: 1) Some adults did not take anthelmintics because their STH infections were asymptomatic; 2) despite receiving anthelmintics, some individuals, including children, chose not to take them; 3) only Ascaris infections provoked concern among participants, as these worms are visible to the naked eye in stool, prompting requests for treatment; 4) The prevalence of ascariasis was below 20%, which was insufficient to motivate widespread participation in PC; 5) economic constraints led people to prioritize meeting their basic daily needs over seeking health interventions. Combined, these factors make the control of STHs through PC challenging, even when we were successful in distributing anthelmintics. In response, our initial focus has shifted towards finding strategies to prevent reinfection. Furthermore, we aim to investigate host factors that may contribute to the low efficacy of ABZ in this context. Addressing both reinfection and ABZ efficacy could significantly enhance the effectiveness of PC in controlling STHs. Currently, our approach remains to treat only those who test positive for STHs, as this at least ensures treatment for those infected and may help mitigate reinfection rates.

Therefore, ABZ treatment was limited to the control group of STH-positive individuals in the community. Due to the pervasive poverty in this area, it is difficult to implement public health education, sanitation, and hygiene interventions. The local population is more focused on meeting their basic needs than on improving sanitation and cleanliness. Poverty, inadequate sanitation and hygiene, ABZ administration being restricted to only positive cases, persistent reinfections, uncured cases, and the challenge of preventing consistently damp soil have all contributed to the ineffectiveness of current control measures. During the driest month of the year, we sought to control hookworms and other STHs, hoping for at least 30 consecutive dry days to potentially dry out the soil. However, achieving this is challenging, as it can rain unexpectedly, even during the dry season, in the tropical rainforest climate of our study area. Notably, a significant drought occurred from February 2014 to March 2015 due to the strongest El Niño events recorded between 2014 and 2016 [14], but were unable to ascertain how this affected STH control. Our goal was to reduce the reinfection rate of STHs by managing the disease during the dry season and assessing the impact of prolonged drought periods on ongoing control efforts without the use of anthelmintics.

## Materials and methods

### Ethics statement

This study received approval from the Ethics Committee in Human Research at Walailak University, Thailand, under approval numbers 12/024 and 15/071. We obtained additional data on the prevalence of STHs for the years 2008 and 2023, under approval numbers 07/009 and 23/028-01, respectively. Data collection specifically related to the prevalence of STHs in school-aged children was approved under number 14/012. Participants reviewed the participant information sheet and provided their written informed consent to participate in the study. For participants under 18, written consent was obtained from their parents.

### Study design

**Fig 1:**
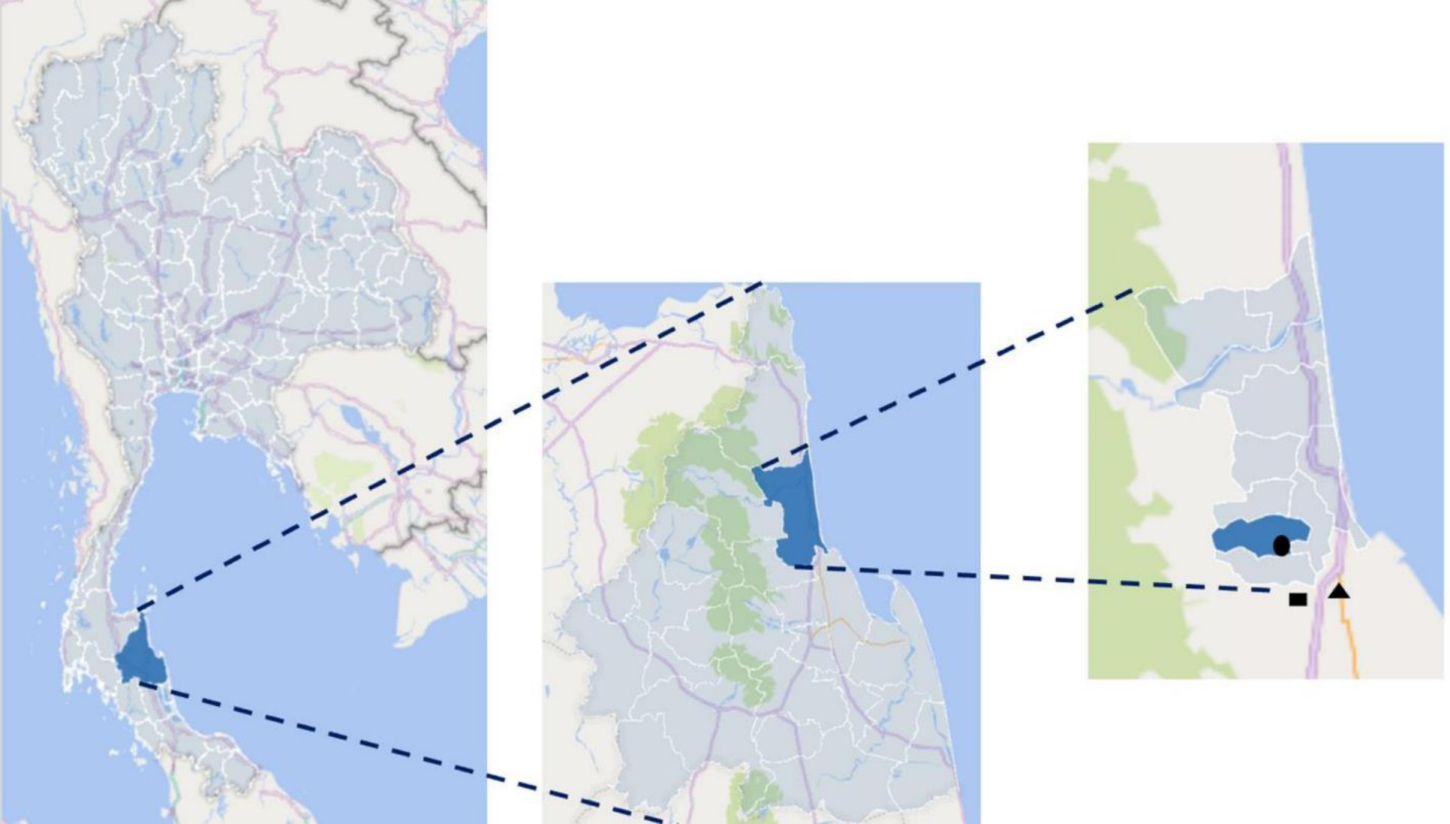
The maps of the study area and two local meteorological stations. The maps show respective (left to right) Nakhon Si Thammarat Province, Thasala District, and Mokhalan Subdistrict (● the study area, ▲ station 0747 and ■ station 0064). The maps were created by Microsoft 365 (Excel) with the available data source from https://github.com/prasertcbs/thailand_gis/blob/main/province/province_region.xlsx and https://github.com/prasertcbs/thailand_gis/blob/main/province/province_simplify/province_simplify.shp for the left map, https://github.com/prasertcbs/thailand_gis/blob/main/amphoe/thailand_province_amphoe.xlsx, and https://github.com/prasertcbs/thailand_gis/blob/main/amphoe/thailand_province_amphoe/thailand_province_amphoe.shp for the middle map, and https://github.com/prasertcbs/thailand_gis/blob/main/tambon/thailand_province_amphoe_tambon_simplify.xlsx and https://github.com/prasertcbs/thailand_gis/blob/main/tambon/shapefiles/Nakhon%20Si%20Thammarat/Nakhon%20Si%20Thammarat.shp for the right map (retrieved on 10 April, 2024).

From March 2012 to June 2016, we conducted a longitudinal study in Village 11, Mokhalan Subdistrict, Thasala District, NST, Thailand, which is located 8 km from our Walailak University laboratory. The geographic coordinates of the study area are 8° 40’ 0" N, 99° 55’ 54" E (Fig 1).

The sample size was calculated according to the formula n = Z^2^P(1-P)/e^2^ due to the uncertain population size in village 11, which also overlapped with other villages.

n, sample size

Z, Z- score for 95% confidence level = 1.96

P, the expected prevalence of STH infection is 74% = 0.74

1-P, the proportion of the population not infected with STHs 26% = 0.26

e, margin of error or confidence interval = 0.05

Participation in this study was entirely voluntary. The following were excluded:

1. Expectant mothers.
2. School-aged children (SAC) at Wat Mokhalan Primary School, the only primary school where some children in the village studied. This exclusion was due to ongoing PC since 2012, though we previously assessed the prevalence in 5-12-year-old SAC in 2008, 2013, and 2017.
3. Children under five years of age.
4. Senior citizens over 75, a rarely encountered age group in this village.

**Fig 2:**
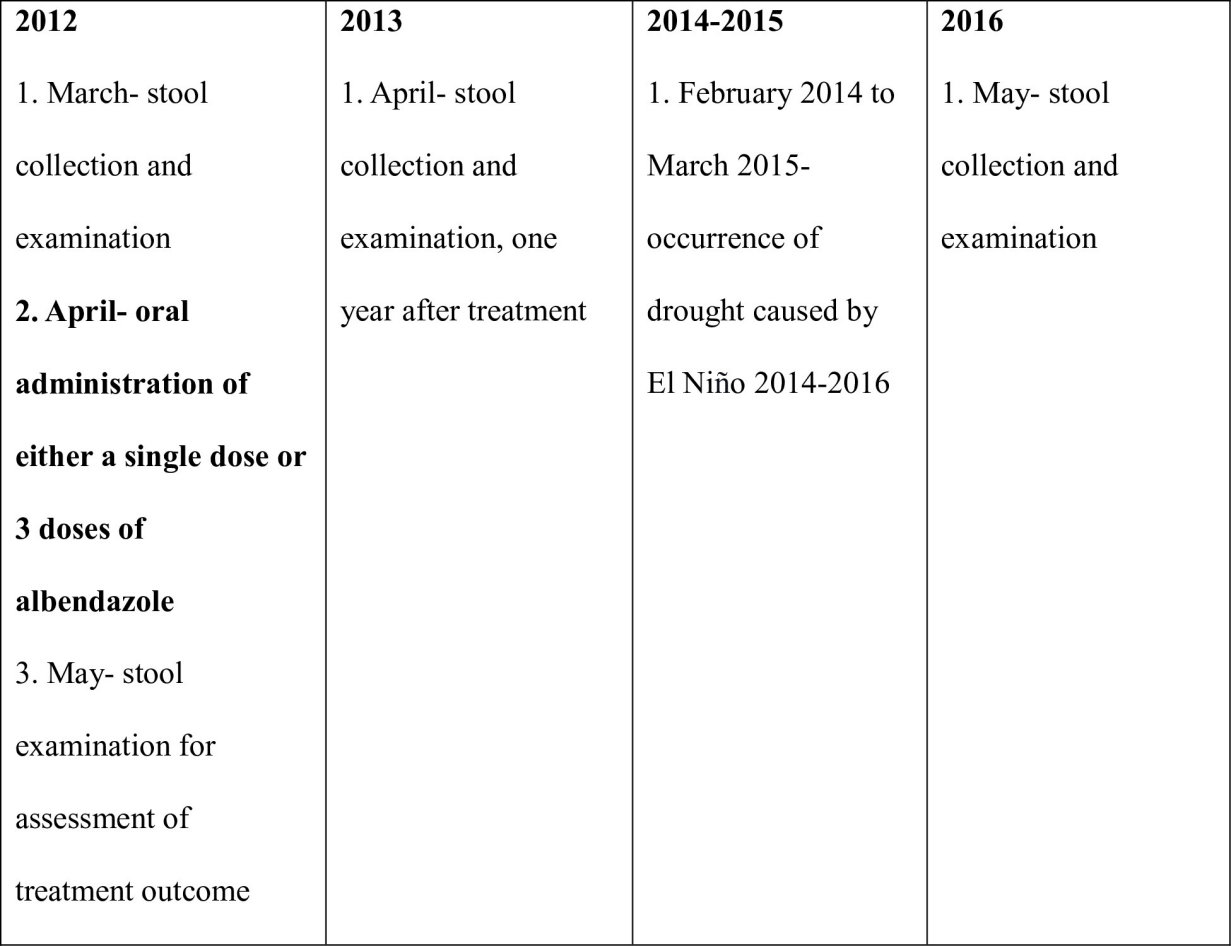
Major study events during 2012–2016.

A total of 299 individuals provided stool samples, which were collected at 6:00 am in a plastic container, stored in a dry box, and transported to the Walailak University laboratory within 10 minutes. The stools were examined within three hours of defecation. Individuals who tested positive for STH eggs received a single 400 mg dose of ABZ. Those testing positive for *S*. *stercoralis* alone, or coinfected with other STHs, were administered three consecutive 400 mg doses of ABZ. Stool samples were collected again three weeks after ABZ administration to assess treatment outcomes. ABZ was administered on April 2, 2012, marking the beginning of the dry season. Rain was recorded for 52 days starting on April 3, 2012. In 2013, stool samples from all 299 participants were collected again and examined for hookworms and other STHs. The final follow-up for 282 participants (17 missing) occurred over the next three years, up to 2016. An unexpected drought, attributed to El Niño from 2014 to 2016, affected the study. Therefore, our longitudinal study from 2012 to 2016 inadvertently included a 14-month-long drought intervention. Major events during this period are summarized in Fig 2.

### Laboratory procedure

Kato-Katz (KK) and agar plate culture (APC) methods were used to detect STHs. Duplicate KK was employed to detect eggs of *A*. *lumbricoides*, *T*. *trichiura*, and hookworm, while triplicate APC was used for detecting *S*. *stercoralis* and hookworm larvae. APC was also used to confirm KK’s ability to detect hookworms, as *Rhabditis*-related eggs can resemble those of hookworms. The KK procedure was as follows [15]: 50 mg of stool was weighed and placed on a glass slide, covered with a cellophane paper soaked overnight in glycerin-phenol-malachite green reagent, spread using a rubber bar, and left at room temperature for 30 minutes. The smears were then examined, and the number of eggs was calculated in duplicate, with the mean number of eggs multiplied by 20 to express the results as eggs per gram of stool. The APC method was performed as follows [16]: 2 grams of stool were placed on a nutrient agar plate and incubated at room temperature for six days. Then, 10 milliliters of 10% formalin were added to the agar surface and the plate was further incubated for 30 minutes. The supernatant was transferred to a 15 ml centrifuge tube and centrifuged at 700 × g for 10 minutes. The pellet was examined for larvae and free-living males and females.

### Rainfall, rainy days, length of dry spells, maximum temperature and relative humidity

From 2006 to 2016, rainfall data in mm for Thailand, the eastern coast of southern Thailand, NST, Thasala district of NST, and two local meteorological stations were obtained from the Thai Meteorological Department (https://www.tmd.go.th) and the National Hydroinformatics Data Center (https://www.thaiwater.net). Rainfall records for Thasala were only available from 2010 to 2014, while data from the two local meteorological stations were available from May 2014 onward. The two local stations, stations 0064 and 0747, located 7 km and 8 km south and southeast of the study area respectively (Fig 1), were chosen as reference stations due to their proximity and the completeness of their data. Data on maximum temperature and relative humidity for 2006–2016 were also sourced from https://www.tmd.go.th. A "dry spell" is defined as a sequence of 15 or more consecutive non-rainy days (with no rain or ≤ 1 mm of rain) during the wet season. The term "dry spell length" refers to the number of consecutive non-rainy days between two precipitation events, which affects soil moisture dynamics. In 2014, a significant dry spell occurred, consisting of 51 consecutive non-rainy days.

The number of days per dry spell from 2011 to 2020 in NST can be accessed at https://www.tmd.go.th. Daily rainfall data for NST in 2012 were also retrieved from this source.

### Statistical analysis

The cure rate (CR) was calculated using Eq 1.

$$CR\;\left(\%\right)=\left(\frac{N_{0}}{N}\right)\times 100$$ Eq 1

where N_0_ is the number of treated participants who were cured, and N is the total number of treated participants. This was calculated for each species.

In addition, the reinfection rate (RR) and new infection rate (NIR) were calculated using Eqs 2 and 3, respectively.

$$Reinfection\;rate\;(RR,\%)=\left(\frac{{\left[N\right]}_{Reinfection}}{{\left[N\right]}_{Infection}}\right)\times 100$$ Eq 2

where N_Reinfection_ is the number of infected individuals who were cured and subsequently became reinfected one (2013) or four (2016) years post-treatment, and N_Infection_ is the total number of participants who were infected at baseline and were initially cured after ABZ treatment.

$$New\;infection\;rate\;(NIR,\%)=\left(\frac{{\left[N\right]}_{New\;infected\;case}}{{\left[N\right]}_{Uninfected\;people}}\right)\times 100$$ Eq 3

where N_New infected case_ is the number of newly infected cases for each species in either 2013 or 2016, N_Uninfected people_ is the number of people uninfected with the corresponding species in 2012.

$$Natural\;cure\;rate\;(NCR,\%)=\left(\frac{{\left[N\right]}_{Cured\;case}}{{\left[N\right]}_{Infection}}\right)\times 100$$ Eq 4

where N_Cured case_ is the number of infected participants who were naturally cured (without ABZ administration), and N_Infection_ is the total number of infected individuals in 2013 who were not treated with ABZ.

The chi-square test was used to compare the differences in prevalence of STHs before and after ABZ administration from 2012 to 2013, and before and after the drought period from 2013 to 2016. Paired t-tests were conducted to assess differences in the intensity of infection before and after both ABZ administration and drought treatment. The chi-square test was also employed to evaluate differences in RRs and NIRs before and after the drought, as well as to compare the NCR of *S*. *stercoralis* and other STHs. A p-value of 0.05 was considered statistically significant. All statistical analyses were performed using IBM SPSS Statistics (version 29.0.0; New York, USA).

## Results

A total of 1,390 people resided in this village, comprising 212 households. Of these, 22 households lacked a latrine (S2 Table). Additionally, human excreta and sewage were not properly disposed of; they were removed from septic tanks and deposited at rubber or palm farms a few kilometers from the village.

From 2006 to 2011, prior to the current study, the average yearly rainfall was 2952.3 mm, and there were 178 rainy days. In contrast, during the study period from 2012 to 2016, the rainfall totaled 2494.3 mm and there were 161 rainy days (S3 Table). The average maximum temperature from 2006 to 2016 ranged from 33.9°C to 35.3°C. However, during April and May from 2014 to 2016, temperatures ranged from 37.0°C to 38.1°C (S4 Table). The average relative humidity from 2006 to 2016 ranged between 80% and 83% (S5 Table).

In 2012, the prevalence of STHs was lowest among participants aged 21–30. There was no significant difference in the prevalence of STHs between sexes across all age groups, except for trichuriasis in the 5–10-year age group, where the prevalence in females was significantly higher than in males (P < 0.05) (Table 1).

**Table 1:** Comparative prevalence of 4 soil-transmitted helminths in males and females by age group in 2012.

				Prevalence of STH infection											
Age	n			Ascariasis			Trichuriasis			Hookworm infection			Strongyloidiasis		
	Total	Male	Female	Total	Male	Female	Total	Male	Female	Total	Male	Female	Total	Male	Female
5–10	17	12	5	11.8	16.7	0	41.2	25	80 ^a^	88	83.3	100	35.3	25	60
11–20	89	44	45	24.7	25	24.4	46.1	50	42.2	79.8	77.3	82.2	43.8	47.7	40
21–30	53	23	30	0	0	0	1.9	4.3	0	28.3	26.1	30	3.8	4.3	3.3
31–40	47	16	31	10.6	6.3	12.9	8.5	6.3	9.7	55.3	50	58.1	19.1	25	16.1
41–50	48	22	26	6.3	0	11.5	39.6	31.8	46.2	72.9	72.7	73.1	25	31.8	19.2
51–60	29	14	15	10.3	7.1	20	10.3	7.1	13.3	68.9	71.4	66.7	20.7	21.4	20
61–70	16	10	6	0	0	0	37.5	40	33.3	68.8	70	66.7	25	30	16.7
Total	299	141	158	12	10.6	13.3	27.1	27.7	26.6	64.5	64.5	64.5	26.1	29.8	22.8

^a^ P < 0.05

The prevalence and intensity of STHs according to infection severity did not significantly differ between sexes, except in the 11-20-year age group. In this group, the intensity of light hookworm infection was significantly higher in females than in males (P < 0.05). Additionally, in the 21-30-year age group, the intensity of light hookworm infection was significantly greater in males than in females (P < 0.05) (S6 Table).

At ages 11–20 years, the prevalence of total single infections was significantly lower than that of total multiple infections (P < 0.001). Conversely, at ages 21–30 and 51–60 years, the prevalence of total single infections was significantly higher than that of total multiple infections (P < 0.00001 and P < 0.01, respectively) (Table 2).

**Table 2:** Prevalence of single and multiple infections of 4 soil-transmitted helminths by age group in 2012.

		Prevalence of single infection					Prevalence of multiple infections							
		Single				Total	Double			Triple			Quartet	Total
Age	n	*Ascaris* (AL)	*Trichuris* (TT)	Hookworm (HW)	*Strongyloides* (SS)		HW+TT	HW+SS	Others	HW+TT +SS	HW+TT +AL	Others	AL+TT+HW+ SS	
5–10	17	0	5.9	35.3	0	41.2	5.9	11.8	0	17.6	5.9	0	5.9	47.1
11–20	89	3.4	2.2	19.1	6.7	31.5 ^b^	11.2	11.2	7.9	10.1	6.7	1.1	7.9	56.2^b^
21–30	53	0	3.8	43.4	3.8	50.9 ^c^	0	5.7	1.9	0	0	0	0	7.5 ^c^
31–40	47	0	2.1	31.9	6.4	40.4	2.1	10.6	6.4	0	2.1	2.1	0	23.4
41–50	48	0	4.2	27.1	0	31.3	18.8	10.4	2.1	12.5	2.1	2.1	0	47.9
51–60	29	3.4	0	48.2	3.4	55.2^a^	0	6.9	3.4	3.4	0	0	6.7	20.7^a^
61–70	16	0	6.3	31.3	0	37.5	18.8	12.5	6.3	6.3	0	0	0	43.8
Total	299	1.3	3.0	31.1	4.0	39.5	8.0	9.7	4.7	6.7	3.0	1.0	3.3	36.5

^a^ P < 0.01

^b^ P < 0.001

^c^ P < 0.00001

### Effect of albendazole administration during the dry season followed by long-term drought on the prevalence and intensity of STHs

**Fig 3:**
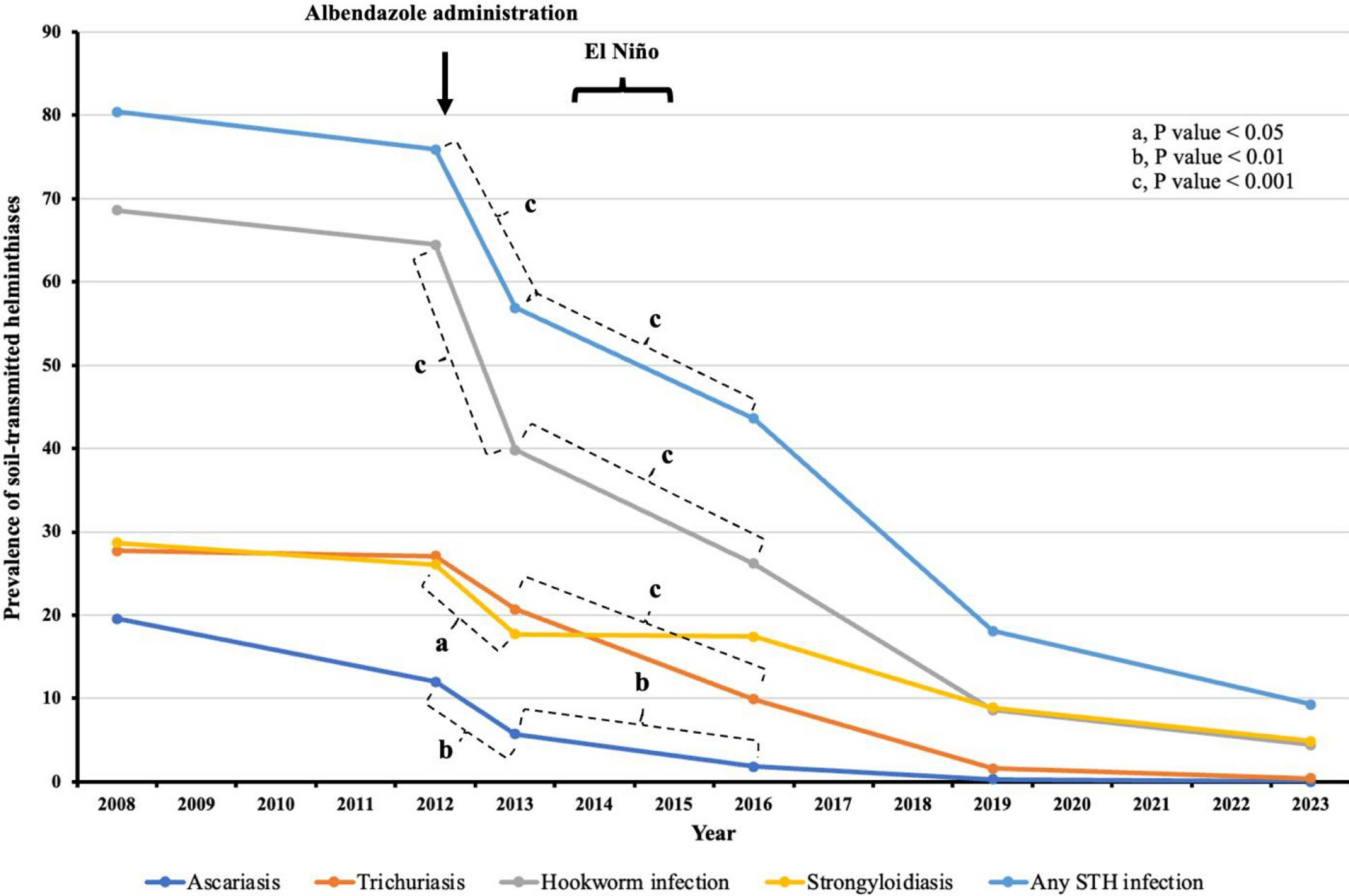
Prevalence of soil-transmitted helminthiases in participants living in village 11 from 2012–2016. A longitudinal study was conducted from 2012 to 2016 with 299 participants. Albendazole was administered in the dry season to STH-positive participants only in 2012. Two follow-ups were done in 2013 and 2016. El Niño 2014–2016 induced a 14-month-long drought from 2014 to 2015. Prevalence of STHs in 2008 (n = 296), 2019 (n = 304), and 2023 (n = 225), were used for comparison before and after the longitudinal study.

Our current study began in 2012 with the administration of ABZ during the dry season, during which there were 38 days without rain, 13 days of slight rain, and only one day of moderate rain (S7 Table). One year later (2013), significant reductions were noted in the prevalence of ascariasis (12.0% vs. 5.7%, P < 0.01), hookworm infection (64.5% vs. 39.8%, P < 0.001), and strongyloidiasis (26.1% vs. 17.7%, P < 0.05) (Fig 3 and S8 Table).

**Fig 4:**
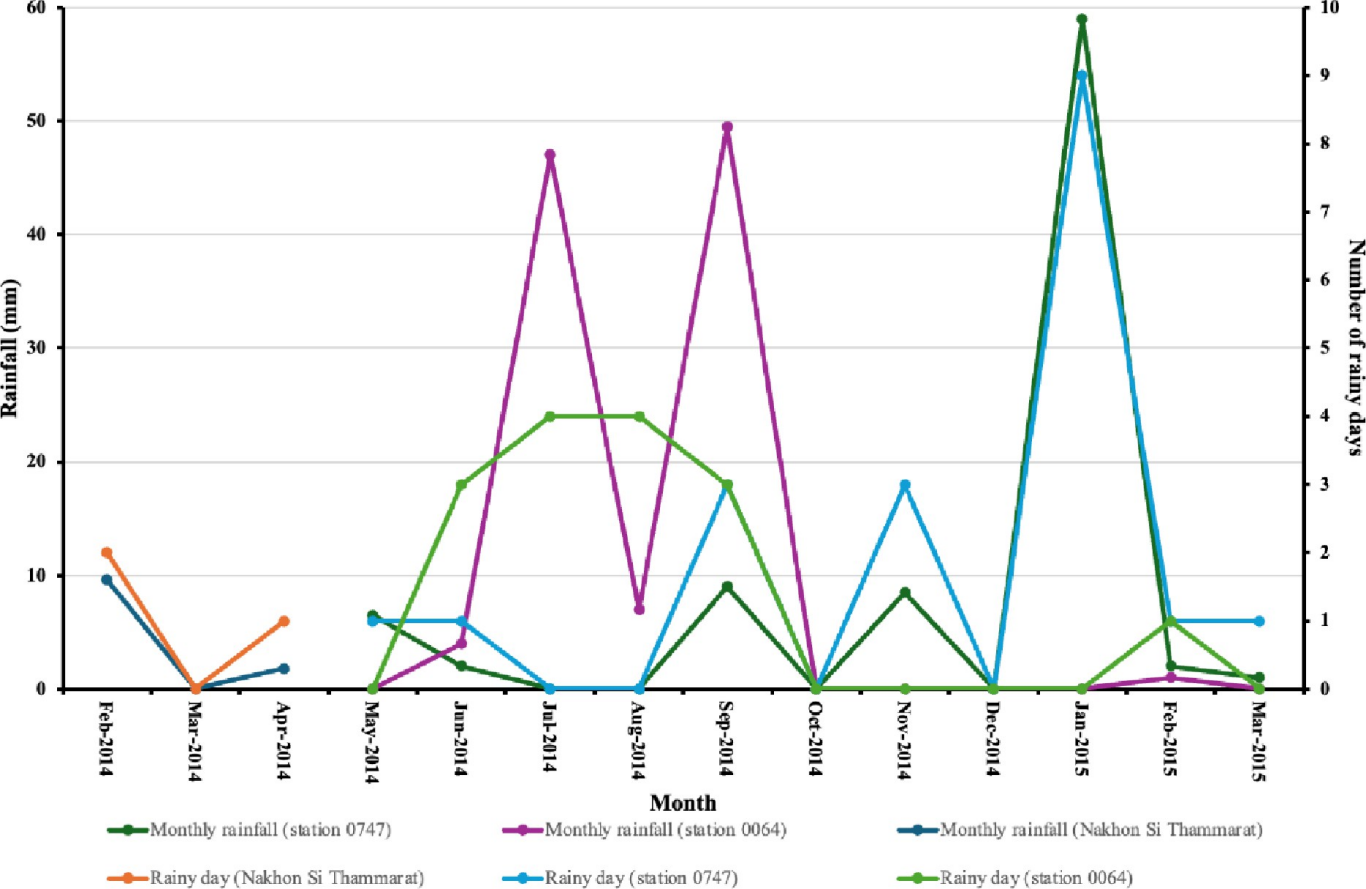
Indicator of drought, rainfall, and rainy days in Nakhon Si Thammarat, stations 0747 and 0064. Monthly rainfall and rainy days in Nakhon Si Thammarat from February to April 2014 were the average measurements of all local meteorological stations. Monthly rainfall and rainy days at local meteorological station 0064 or station 0747 were measured only at station 0064 or station 0747.

The intensity of ascariasis and hookworm infection also decreased significantly (6255 ± 4108.9 vs. 6663.5 ± 12138.7, P < 0.01; 740.3 ± 561.2 vs. 394.9 ± 198.2, P < 0.001) (S8 Table). However, the prevalence and intensity of trichuriasis remained unchanged, likely due to the low efficacy of albendazole against this infection. Droughts, triggered by El Niño episodes, occurred between 2014 and 2015. From February-April 2014 to May-December 2014 (Fig 4 and S3, S9 and S10 Tables), there was minimal rainfall (1.6, 0, and 3 mm/month in NST; 0, 4, 47, 7, 49.5, 0, and 0 mm/month at local meteorological station 0064; 6.5, 2, 0, 9, 0, and 8.5 mm/month at local meteorological station 0747).

**Fig 5:**
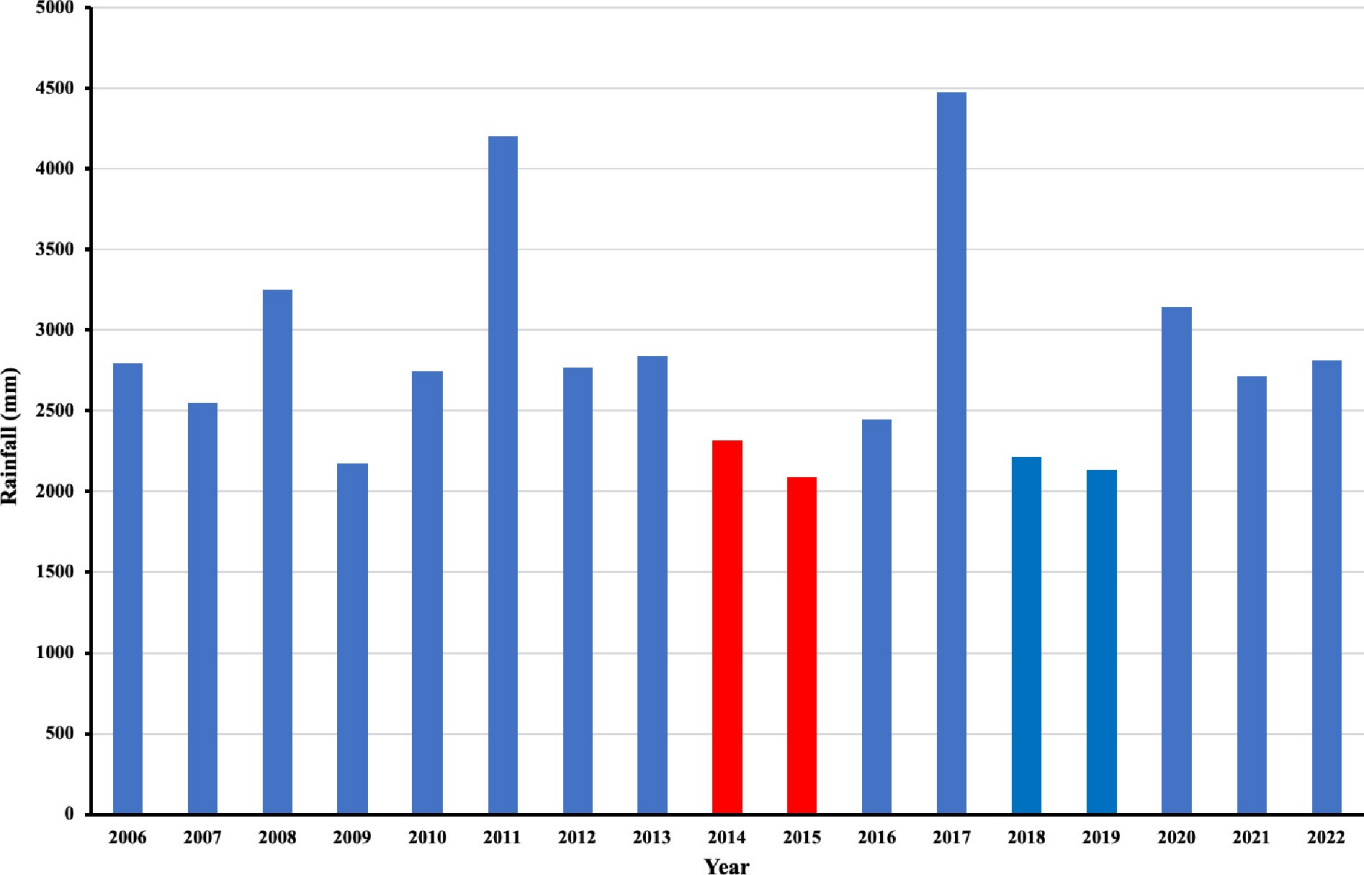
Rainfall in Nakhon Si Thammarat Province from 2006 to 2022. The strongest El Niño event in 2014–2016 induced a long drought in 2014–2015.

Additionally, there was a significant lack of rainy days during these periods (3 of 89 days in NST, 8 of 183 days at station 0747, and 14 of 214 days at station 0064). In 2015, limited rainfall continued from January to March (59, 2, and 1 mm at station 0747; 0, 1, and 0 mm at station 0064) with few rainy days (11 of 90 days at station 0747 and 1 of 90 days at station 0064). Yearly rainfall from 2006–2022 in NST Province which was an average of approximately 100 local meteorological stations in the province was shown (Fig 5). El Niño also led to 51 dry spell days in 2014 and 31 in 2015 (S1 Table).

In 2016, after a three-year follow-up period without treatment, there was a continued decrease in the prevalence of ascariasis and hookworm infection, from 5.7% to 1.8% (P < 0.01) and from 39.8% to 26.2% (P < 0.001), respectively. The intensity of these infections also decreased significantly (from 6664 to 1640, P < 0.05; and from 395 to 205, P < 0.001, respectively). Notably, the previously unchanged prevalence of trichuriasis significantly decreased (from 20.7% to 9.9%, P < 0.001). However, the prevalence of strongyloidiasis remained unchanged. Additionally, the prevalence of infection with any STH continued to decrease following ABZ administration in 2012 and in 2016 (P < 0.001) (Fig 3 and S8 Table).

Additionally, supporting data from school-aged children aged 5–12 who had received PC since 2012 are detailed in the S11 Table. Following the drought, similar to the broader community, there was a significant decrease in the prevalence and intensity of ascariasis, trichuriasis, and hookworm infection (from 3.9% to 0%, 15.4% to 5.3%, P < 0.05; and from 42.3% to 10.7%, P < 0.00001, respectively). While the prevalence of strongyloidiasis did not significantly decrease, it showed a downward trend, moving from 10.3% (8/78) in 2013 to 4.0% (3/75) in 2017.

Surveillance data from the community in 2019 indicated a continuing decline in the prevalence of STHs, including strongyloidiasis. Of the 304 randomly sampled participants, 72 were children aged 3–12. Notably, only 3 children (4.2%) tested positive for hookworm eggs, while tests for *Ascaris* eggs, *Trichuris* eggs, and *Strongyloides* larvae were negative across the board. By 2023, data showed a persistent decrease in the prevalence of STHs among adults over 17 years, with ascariasis eliminated, trichuriasis approaching zero, and lower incidences of hookworm infection and strongyloidiasis (S8 Table).

### Albendazole treatment outcomes versus drought in the treatment of STHs excluding strongyloidiasis

**Fig 6:**
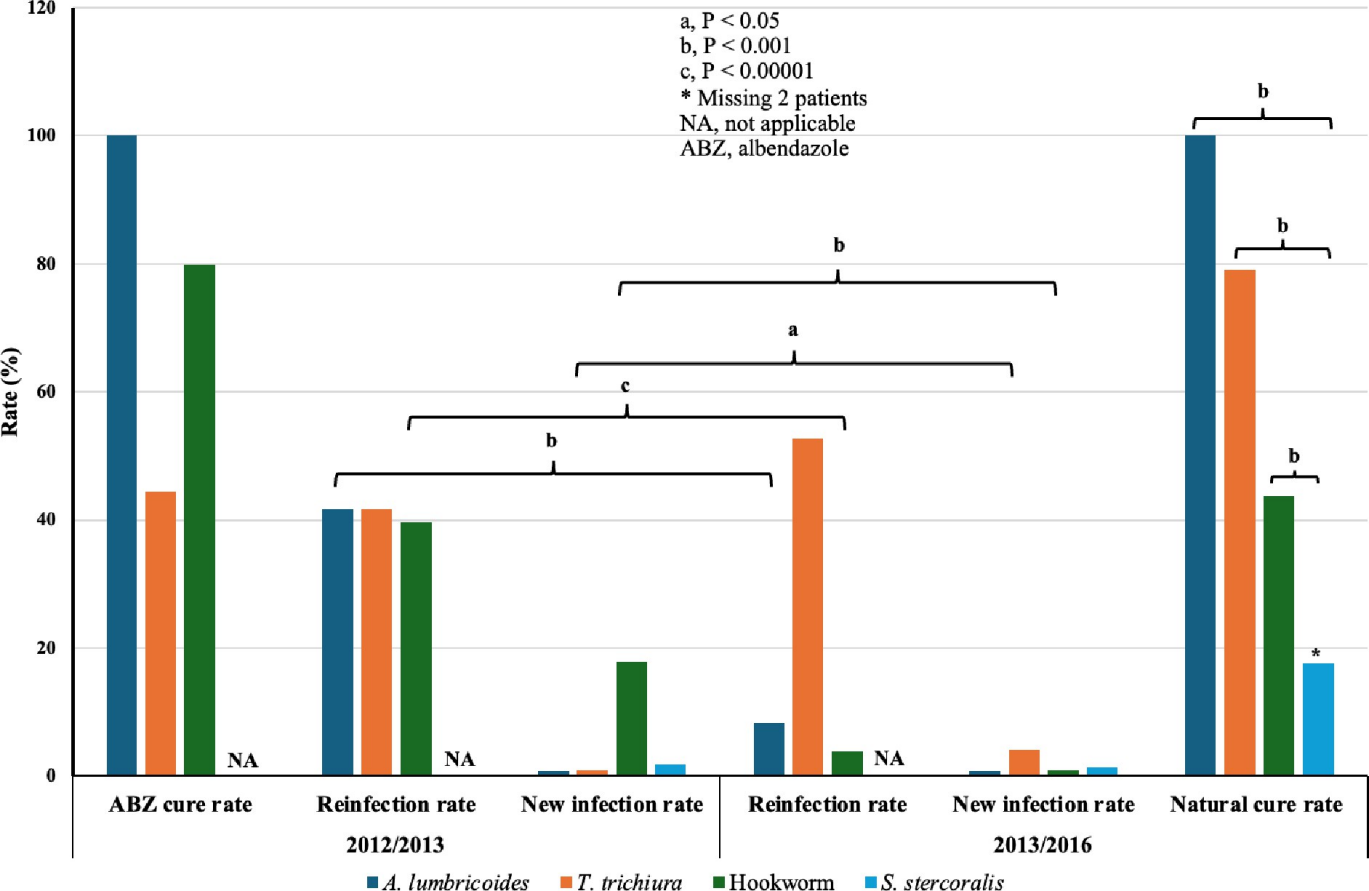
Response of soil-transmitted helminths to albendazole (2012/2013) and drought (2013/2016). In 2012, ABZ cure rate was calculated three weeks post-treatment. Reinfection and new infection rates were calculated in 2013 and 2016, one or four years post-treatment. The natural cure rate was calculated in 2016, three years after 2013, and one-year post-drought.

The ABZ cure rates (CRs) for *A*. *lumbricoides*, *T*. *trichiura*, and hookworm were 100%, 44.4%, and 79.8%, respectively (S12 Table). Additionally, ABZ did not cure 45 patients with *Trichuris* and 39 with hookworm infections. One year after ABZ administration, the reinfection rates (RRs) for ascariasis, trichuriasis, and hookworm infection were 41.7%, 41.7%, and 39.6%, respectively (Fig 6).

The new infection rates (NIRs) for these helminthiases and strongyloidiasis were 0.76%, 0.92%, 17.9%, and 1.8%, respectively. Post-drought, the prevalence of ascariasis and hookworm infection significantly decreased, with RRs of 8.3% (P < 0.001) and 3.9% (P < 0.00001), respectively, while the RR for trichuriasis did not significantly change (P > 0.05). The NIR for trichuriasis significantly increased (P < 0.05), whereas the NIR for hookworms significantly decreased (P < 0.001). The natural cure rates (NCRs) in 2016 for *T*. *trichiura* and hookworm were 79% (49/62) and 43.7% (52/119), respectively, while that of ascariasis was 100% (17/17). Of the 49 naturally cured trichuriasis cases, seven were reinfected, two were newly infected, and 40 were uncured. The 52 naturally cured hookworm cases included 24 reinfected, 15 newly infected, and 13 uncured cases. Among the 17 naturally cured cases of ascariasis, 15 were reinfected, and 2 were new infections. Despite a low CR, the prevalence of trichuriasis markedly decreased. Notably, the overall NCR for strongyloidiasis was significantly lower than for the other three STHs (P < 0.001) (Fig 6 and S12 Table).

## Discussion

In 2013, one year after administering albendazole in the dry season, the prevalence of trichuriasis remained unchanged due to its low CR of 44%, attributed to the limited efficacy of albendazole. In contrast, the prevalence of ascariasis declined significantly, supported by a high CR of 100%. Furthermore, the RRs and NIRs for both parasites showed no significant differences. It is well-documented that a single 400 mg dose of albendazole is highly effective against ascariasis but less so against trichuriasis [17]. The development of infective *Ascaris* eggs requires longer periods than those for *Trichuris*, potentially increasing exposure to *Ascaris* eggs when temperatures exceed 37°C [18]. This prolonged exposure to high temperatures, coupled with a lower baseline prevalence, shorter lifespan, and higher cure rate, likely contributed to the more rapid reduction in *Ascaris* prevalence compared to *Trichuris*. Furthermore, over the decade from 2005 to 2015, the overall prevalence of ascariasis in Africa decreased by 10%, while trichuriasis saw a modest reduction of only 2% [19]. The prevalence of hookworm infections also decreased, due to an 80% CR and a RR similar to that of trichuriasis. Moreover, the prevalence of strongyloidiasis decreased, potentially due to the administration of three consecutive doses of albendazole, despite its lower efficacy compared to ivermectin [20].

In 2016, one year following a drought, there was a noted decrease in the prevalence of ascariasis, trichuriasis, and hookworm infections. However, the prevalence of strongyloidiasis remained unchanged, attributed to its low NCR likely due to the drought’s limited impact. *S*. *stercoralis* is capable of reproducing within the host, allowing it to survive for extended periods, up to 65 years [21]. Unlike other STHs, 5–10% of the rhabditiform larvae of *S*. *stercoralis* in stool can develop into infective larvae within 24 hours under the high temperatures in our study area [22]. This rapid development provides *S*. *stercoralis* larvae with as greater opportunity to infect new hosts before succumbing to desiccation compared to other STHs. Additionally, rhabditiform larvae fail to develop if submerged in water [23] or if the stool is diluted more than 160 times [24], due to insufficient air or the depletion of essential stool nutrients, respectively. Consequently, *S*. *stercoralis* does not thrive in conditions of rainfall as do other STHs. In contrast, while a dry environment outside the host may generally favor the survival of *S*. *stercoralis*, it can be detrimental to other STHs. Notably, the El Niño phenomenon contributed to a prolonged drought, followed by dry soil conditions exacerbated by climate change, and possible improvement in sanitation and hygiene practices, which negatively impacted the transmission of *S*. *stercoralis*. Three years after this study, the prevalence of *S*. *stercoralis* decreased, and continued to decline over the following year. Subsequently, *S*. *stercoralis* became the predominant species relative to other STHs. The prevalence of ascariasis, trichuriasis, and hookworm infections decreased following the drought, driven by increased NCRs for these worms, along with decreased RRs of ascariasis and hookworm infections, and reduced NIRs of hookworm. However, the RR of trichuriasis remained unchanged, and its NIR increased, indicating a more widespread presence of *Trichuris* compared to *Ascaris* in the study area (Table 1). Additionally, temperatures below 37°C promote the embryonation of *Trichuris* eggs, facilitating their transmission [17].

Wet soil can facilitate the development and survival of STHs, especially in rural areas where roads paved with concrete and cement are rare. When infected human stool or excreta are deposited on wet soil, which acts much like a nutrient-rich medium, the eggs can evolve into infective larvae capable of surviving in the environment for weeks or months, depending on the species [18]. Conversely, placing stool on dry soil can disrupt the development of hookworm rhabditiform larvae, ultimately halting their growth [7]. Drought conditions cause rapid evaporation of water from stool and hinder water absorption from stool placed on dry soil, disrupting the life cycle of STHs. Under such conditions, all stages of hookworm larval development cease within a few days [7]. Moreover, while the eggshells of *A*. *lumbricoides* and *T*. *trichiura* may shield the developing larvae inside from desiccation in dry stool, they cannot protect against high temperatures (> 37°C) [6]. Additionally, the absence of water during droughts prevents the movement of infective eggs or larvae from one area to another, further interrupting the transmission of STHs. This as well as improvement in sanitation and hygiene practices results in a decrease in RRs and NIRs. The NCRs for *T*. *trichiura*- and *A*. *lumbricoides*-infected participants were higher than those for hookworm-infected individuals due to the longer lifespan of hookworms. The life spans of *A*. *lumbricoides*, *T*. *trichiura*, and hookworm are 1–2, 1–2, and 3–4 years, respectively [18]. While most hookworms have a lifespan of 3–4 years, certain species of hookworms can survive for up to 18 years [25]. The development of larvae, both inside and outside the egg, was notably diminished during periods of drought [26]. *N*. *americanus* and *Ancylostoma* species generally require 3–10 days to develop into infective larvae. In contrast, the eggs of *A*. *lumbricoides* and *T*. *trichiura* need 28–84 and 10–30 days, respectively, to become infective [18]. Furthermore, the NCRs for reinfected and newly infected cases of *Ascaris* (17/17), hookworm (39/52), and *Trichuris* (9/49) suggest possible influence from the drought and potentially from improved hygiene practices, rather than solely from the administration of ABZ in the dry season.

STH control during the dry season may have contributed to a decrease in the prevalence of STHs, with the exception of trichuriasis. However, the prevalence likely increased subsequently because the sample size was only 299 out of the total 1,390 people in the village, leaving most positive STH cases untreated and acting as large reservoirs of infection. A previous intervention in Ghana during the dry season resulted in a significant decrease in the prevalence of *Esophagostomum bifurcum* and hookworm infections, from 53% to 5.4% and from 86.9% to 36.8%, respectively, after two rounds of mass treatment with a single oral dose of ABZ. The prevalence of both infections further decreased to 0.8% and 23.4%, respectively, after four rounds of treatment [27]. It is important to note that this was achieved through MDA, whereas our study involved only the oral administration of albendazole to participants who tested positive. Furthermore, in the absence of the El Niño-induced long drought, and/or the improvement in sanitation and hygiene practices, the prevalence in 2016 should have mirrored that of 2012. Additionally, in another study area located on the seashore at Village 4, Thasala Subdistrict, where only trichuriasis is prevalent. The control area located in the village is composed of three houses with roofs linked together, so sunlight cannot expose the narrow sandy ground. Three families have lived here. All of them are relatives with twenty members: seven adults and thirteen children ages two to seven years. All were infected with *T*. *trichiura*. People here wash foot before entering their houses. This characteristic makes the soil wet all the time. Young children frequently defecated on the soil. Thus, the life cycle of *T*. *trichiura* has been completely maintained. In 2016, one year post-drought, the prevalence of trichuriasis in the village with dry sandy soil was significantly decreased whereas in the control area with artificially wet sandy soil did not decrease (S13 Table). It reflected that the prevalence of trichuriasis in the village would not decrease in the presence of rainfall.

In 2016, we wondered why three children previously infected with hookworms and uncured by albendazole had been naturally cured despite frequently playing football barefoot. After that, the children have not been reinfected by any STHs anymore. We observed that the dry playground or soil has often been seen from 2014 to the present due to climate change. While the number of households lacking latrines decreased, excreta disposal was still poor. Excreta from some households was removed from septic tanks and deposited at rubber or palm farms a few kilometers from the village, however, some households deposited excreta near their homes. The excreta samples collected from thirty households were examined and 60% were positive for hookworm infective larvae using agar plate culture, and 10% were positive for *Trichuris* eggs. The excreta disposal here remains unchanged because of a lack of a disposal system. However, the life cycle of STHs in the excreta ceased under the dry soil conditions in the village (7). Hygiene might be improved in some households not exceeding 20% of the population which was not enough to cease the transmission of hookworms. Furthermore, hygiene depends on the individual. In addition, we followed two isolated areas (not included in the present study) because there were three ABZ uncured cases of hookworm infection over there, one had fifteen members in three households and the other had thirteen members in a household. We observed that both had no latrine, people walked barefoot when going outside, and drank unboiling groundwater. Post-drought the prevalence of STHs decreased despite poor WASH status (S14 and 15 Tables). Hence, we touched on drought from 2014 to 2015 likely to contribute to the successful STH control.

Approximately 30 El Niño events have occurred since 1990, with the three most significant recorded in 1982–1983, 1997–1998, and 2014–2016 [28]. Additional occurrences were noted from 2018 to 2019, and the phenomenon is ongoing in 2023. The El Niño event of 2014–2016 led to drought across the southern region and throughout Thailand. Droughts associated with El Niño events may contribute to a decrease in the prevalence of STHs in both controlled and uncontrolled areas. Two national surveys on the prevalence of STHs in Thailand conducted in 2009 [9] and 2019 [10] indicated a decline in the overall prevalence of hookworm infection and opisthorchiasis, from 6.5% and 8.7% to 4.5% and 2.2%, respectively. In southern Thailand, the prevalence of ascariasis, trichuriasis, and hookworm infection also decreased from 1.7%, 3.9%, and 15.8% to 0.7%, 2.2%, and 9.8%, respectively [9,10]. Additionally, in 2016, two studies involving schoolchildren and adults, and another study of elderly individuals in 2019 in Thasala, NST, Thailand, recorded prevalence rates of ascariasis, trichuriasis, hookworm infection, and strongyloidiasis at 0, 0.3, 10.7, and 0%; 0, 0.3, 8.0, and 0.9%; and 0, 2.1, 10.9, and 3.4%, respectively [29–31]. The studies also indicated that participants remained at a high risk of STH infection due to several factors: open defecation (7.5–18.5%); absence of hand washing before meals (15.5–25.1%); walking barefoot (2.2–54.2%); drinking unboiled or unfiltered water among schoolchildren (50%); and consuming unwashed vegetables (58–94%). Despite the decreasing prevalence and intensity of STHs, similar poor hygiene conditions were observed in our study area. Notably, in 2012, participants aged 21–30 years exhibited the lowest prevalence of STHs (Table 1), possibly due to better self-care associated with young adulthood. Over time, we expected that individuals aged 11–20 years may improve their personal hygiene practices as they mature. Nevertheless, public health education remains essential for enhancing sanitation and hygiene practices to ensure the sustainable control of STHs in southern Thailand.

The first limitation of the present study is the lack of a questionnaire on the hygiene of the participants. However, in most cases, we visited participants’ households and provided knowledge on preventing STHs. Additionally, positive participants, particularly homemakers, often requested information on preventing reinfection after treatment, and we provided guidance accordingly. Despite improvements, as evidenced by the reduction from 22 households without latrines in 2012 to just eight in 2016, people still use excreta from septic tanks as fertilizer, which might impact the prevalence of STHs. The second limitation concerns the small sample size of 299, which might affect the power of statistical analysis and may only be representative of a portion of the population. The third limitation is that parameters affecting infection were not controlled for in the analysis, and the treatment’s effect during the dry season alone can have a substantial impact, as STHs can be controlled when the prevalence falls below a certain threshold. The fourth limitation involves the administration of albendazole to STH-positive participants during the dry season, specifically to manage hookworm reinfection. If there is no prolonged drought, we plan to apply this new knowledge in subsequent preventative campaigns. Lastly, the study combined data from single and multiple infections to calculate the metric of interest. A previous study indicated a positive association between *Trichuris*, hookworm, and *Ascaris*. Egg depositions for *Ascaris* and hookworm were lower in single infections compared to co-infections [32]. However, all *Ascaris* cases were cured, regardless of whether they were single or co-infections. Furthermore, 109 participants were co-infected with ten patterns, making statistical analysis challenging due to the small sample size for each pattern.

## Conclusion

A control effort in the dry season followed by a 14-month drought induced by El Niño during 2014–2016, along with some improvement in sanitation and hygiene, led to decreases in the prevalence and intensity of STHs, excluding *S*. *stercoralis*. There were decreases in both RR and NIR for hookworms, a decrease in the RR for *A*. *lumbricoides*, and increases in the NCRs for *A*. *lumbricoides*, *T*. *trichiura*, and hookworm. These changes contributed to reduced prevalence and intensity levels. Our findings suggest that control of STHs should be initiated at the beginning of the dry season.

## Supporting information

S1 Table Rainfall in mm in Thailand, the eastern coast of southern Thailand, Nakhon Si Thammarat (NST) Province, Thasala District, and 2 local meteorological stations and dry spell length days in NST Province. (DOCX)

S2 Table Demographic characteristics of village 11 in the Moklalan Subdistrict of Thasala District, Nakhon Si Thammarat, southern Thailand 2012. (DOCX)

S3 Table Rainfall and rainy days in Nakhon Si Thammarat during 2006–2016. (DOCX)

S4 Table Maximum temperature in Nakhon Si Thammarat during 2006–2016. (DOCX)

S5 Table Relative humidity in Nakhon Si Thammarat during 2006–2016. (DOCX)

S6 Table Prevalence and intensity of 3 soil-transmitted helminths by severity of infection, sex, and age in 2012. (DOCX)

S7 Table Rainfall and rainy days after albendazole intake for 52 days from April 3 to May 24, 2012. (DOCX)

S8 Table Prevalence and intensity (egg/gram of stool) of 4 soil-transmitted helminthiases in participants who received albendazole in 2012 only, living in village 11 and in surrounding areas from 2012–2016. (DOCX)

S9 Table Monthly rainfall and rainy days were measured by station 0747 in Ban Tha Ngam, one of the stations nearest to Village 11 of Mokhalan. (DOCX)

S10 Table Monthly rainfall and rainy days were measured by station 0064 Ban Nai Tub, one of the stations nearest to Village 11 of Mokhalan, and with the most complete data availability since 2014. (DOCX)

S11 Table Prevalence and intensity (egg/gram of stool) of 4 soil-transmitted helminthiases in school-aged children (SAC) at Wat Mokhalan primary school who had received preventive chemotherapy (either a single dose of 400 mg albendazole or 500 mg mebendazole) since 2012. (DOCX)

S12 Table Response of STHs to albendazole 3 weeks after albendazole administration and to drought one year after long-lasting drought. (DOCX)

S13 Table Comparative prevalence of trichuriasis between a control area where wet soil has been artificially maintained and in other areas with dry sandy soil of Village 4 Thasala Subdistrict located at the seashore 2013–2018. (DOCX)

S14 Table Prevalence and intensity (egg/gram, larva/gram of stool) of 4 soil-transmitted helminths in people in isolated area 1 of Village 11, 2008–2019. (DOCX)

S15 Table Prevalence and intensity (egg/gram, larva/gram of stool) of 2 soil-transmitted helminths in people in isolated area 2 of Village 11, 2008–2019. (DOCX)

S1 Data Raw data for S8 Table. (XLSX)

S2 Data Raw data for S12 Table. (XLSX)

S3 Data Numerical value for graphs Figs 3 and 5. (XLSX)
