# Supplementary material for: The enhancive effect of the 2014–2016 El Niño-induced drought on the control of soil-transmitted helminthiases without anthelmintics: A longitudinal study
Source: PLoS Negl Trop Dis. 2024 Jul 12;18(7):e0012331. doi: 10.1371/journal.pntd.0012331 (PMC11268648; doi:10.1371/journal.pntd.0012331)
Supplement: S1 Table — (DOCX) [file pntd.0012331.s001.docx]

**S1 Table. Rainfall in mm in Thailand, the eastern coast of southern Thailand, Nakhon Si Thammarat (NST) Province, Thasala District, and 2 local meteorological stations and dry spell length days in NST Province.**

|  | **Rainfall in mm** |  |  |  |  |  | **No. of DSL**  **days** |  |
| --- | --- | --- | --- | --- | --- | --- | --- | --- |
| **Year** | **Thailand** | **Eastern coast of southern Thailand** | **NST** | **Thasala District**  **NST** | **STN 0064** ^a^ | **STN 0747** ^a^ | **NST**  **(dry season)** | **NST**  **(wet season)** |
| 2006 | 1692.5 | 1785 | 2790.9 | ND | ND | ND | ND | ND |
| 2007 | 1641.7 | 1964 | 2546.7 | ND | ND | ND | ND | ND |
| 2008 | 1751.4 | 2062 | 3251.4 | ND | ND | ND | ND | ND |
| 2009 | 1609.8 | 1786 | 2175.5 | ND | ND | ND | ND | ND |
| 2010 | 1650.5 | 2058 | 2747.9 | 2225.9 | ND | ND | ND | ND |
| 2011 | 1947.9 | 2496 | 4201.6 | 3168.1 | ND | ND | 11 | 7 |
| 2012 | 1681.7 | 2032 | 2771.5 | 1907.8 | ND | ND | 6 | 12 |
| 2013 | 1764.4 | 2050 | 2840.6 | 1979.2 | ND | ND | 13 | 6 |
| 2014 | 1520.4 | 1878 | 2318.5 | 1728.9 | 107.5 | 26 | 51 | 1 |
| 2015 | 1419.7 | 1648 | 2091.5 | ND | 1306 | 910 | 31 | 2 |
| 2016 | 1716.1 | 1911 | 2449.3 | ND | 2179 | 561.5 | 29 | 4 |
| 2017 | 2017.1 | 2767 | 4478.4 | ND | 4757 | 3996 | 17 | 6 |
| 2018 | 1660.9 | 1988 | 2218.8 | ND | 1733 | 1810.5 | 11 | 5 |
| 2019 | 1343.4 | 1611 | 2132.2 | ND | 195 | 35 | 38 | 5 |
| 2020 | 1527.3 | 2154 | 3141.6 | ND | 216.5 | 91.5 | 11 | ND |
| 2021 | 1759.3 | 2083 | 2718 | ND | 670 | 1011.5 | ND | ND |

^a^ Reference station in this study

ND, no data

DSL, dry spell length

STN, Local meteorological station

NST, Nakhon Si Thammarat Province

The data were retrieved from https://www.thaiwater.net/weather/rain
