# Supplementary material for: The enhancive effect of the 2014–2016 El Niño-induced drought on the control of soil-transmitted helminthiases without anthelmintics: A longitudinal study
Source: PLoS Negl Trop Dis. 2024 Jul 12;18(7):e0012331. doi: 10.1371/journal.pntd.0012331 (PMC11268648; doi:10.1371/journal.pntd.0012331)
Supplement: S2 Table — (DOCX) [file pntd.0012331.s002.docx]

**S2 Table. Demographic characteristics of village 11 in the Moklalan Subdistrict of Thasala District, Nakhon Si Thammarat, southern Thailand 2012.**

|  | **Village 11 of Moklalan** |
| --- | --- |
| No. of household | 212 |
| No. of population | 1390 |
| No. of mosque | 1 |
| No. of the preschool center | 0 |
| No. of Subdistrict Health Promotion Hospital | 1 |
| No. of households without latrines | 22 |
| Source of water supply | Groundwater |
| Road | Earthen road  gravel road |
| Soil type | Sandy loam |

The data were retrieved from https://www.mokhalan.go.th
