# Supplementary material for: The enhancive effect of the 2014–2016 El Niño-induced drought on the control of soil-transmitted helminthiases without anthelmintics: A longitudinal study
Source: PLoS Negl Trop Dis. 2024 Jul 12;18(7):e0012331. doi: 10.1371/journal.pntd.0012331 (PMC11268648; doi:10.1371/journal.pntd.0012331)
Supplement: S4 Table — (DOCX) [file pntd.0012331.s004.docx]

**S4 Table. Maximum temperature in Nakhon Si Thammarat during 2006-2016**

|  | **Maximum temperature Celsius during 2006-2016** | | | | | | | | | | | | |
| --- | --- | --- | --- | --- | --- | --- | --- | --- | --- | --- | --- | --- | --- |
| **Year** | **Jan** | **Feb** | **Mar** | **Apr** | **May** | **Jun** | **Jul** | **Aug** | **Sep** | **Oct** | **Nov** | **Dec** | **Aver** |
| 2006 | 32.0 | 32.2 | 34.4 | 35.0 | 34.5 | 34.8 | 35.6 | 35.4 | 33.8 | 33.7 | 32.6 | 32.5 | 33.9 |
| 2007 | 32.0 | 33.2 | 35.0 | 35.0 | 35.7 | 35.8 | 36.8 | 37.0 | 35.5 | 34.5 | 31.5 | 31.9 | 34.5 |
| 2008 | 32.0 | 32.7 | 34.2 | 35.9 | 35.7 | 35.7 | 36.2 | 35.8 | 36.0 | 34.8 | 34.0 | 31.2 | 34.5 |
| 2009 | 31.6 | 34.1 | 34.8 | 37.0 | 36.0 | 37.0 | 36.3 | 36.5 | 36.5 | 34.4 | 33.7 | 32.5 | 35.0 |
| 2010 | 32.8 | 34.5 | 35.2 | 36.7 | 37.5 | 36.5 | 36.3 | 35.9 | 35.5 | 35.0 | 31.7 | 32.0 | 35.0 |
| 2011 | 31.6 | 33.6 | 33.6 | 34.1 | 36.6 | 36.2 | 35.9 | 36.4 | 35.3 | 34.4 | 34.0 | 32.2 | 34.5 |
| 2012 | 31.9 | 33.3 | 35.5 | 36.3 | 37.8 | 37.2 | 37.5 | 38.5 | 35.5 | 34.0 | 33.7 | 32.5 | 35.3 |
| 2013 | 32.8 | 32.6 | 36.2 | 37.5 | 36.2 | 36.5 | 36.0 | 36.3 | 36.0 | 35.4 | 33.1 | 31.7 | 35.0 |
| 2014 | 32.5 | 32.5 | 35.5 | 37.0 | 38.0 | 36.7 | 37.2 | 36.2 | 36.0 | 34.5 | 33.6 | 33.5 | 35.3 |
| 2015 | 31.6 | 32.4 | 34.9 | 37.0 | 37.1 | 37.1 | 37.0 | 36.2 | 35.1 | 35.0 | 33.2 | 32.9 | 35.0 |
| 2016 | 33.7 | 32.5 | 34.6 | 38.0 | 38.1 | 36.5 | 35.5 | 36.0 | 36.2 | 35.2 | 33.6 | 33.5 | 35.3 |

The data were retrieved from https://www.tmd.go.th, November 29, 2023
