# Supplementary material for: The enhancive effect of the 2014–2016 El Niño-induced drought on the control of soil-transmitted helminthiases without anthelmintics: A longitudinal study
Source: PLoS Negl Trop Dis. 2024 Jul 12;18(7):e0012331. doi: 10.1371/journal.pntd.0012331 (PMC11268648; doi:10.1371/journal.pntd.0012331)
Supplement: S5 Table — (DOCX) [file pntd.0012331.s005.docx]

**S5 Table. Relative humidity in Nakhon Si Thammarat during 2006-2016**

|  | **Relative humidity during 2006-2016** | | | | | | | | | | | | |
| --- | --- | --- | --- | --- | --- | --- | --- | --- | --- | --- | --- | --- | --- |
| **Year** | **Jan** | **Feb** | **Mar** | **Apr** | **May** | **Jun** | **Jul** | **Aug** | **Sep** | **Oct** | **Nov** | **Dec** | **Aver** |
| 2006 | 84 | 83 | 80 | 82 | 84 | 83 | 79 | 79 | 83 | 84 | 86 | 84 | 83 |
| 2007 | 84 | 80 | 79 | 81 | 84 | 81 | 79 | 76 | 80 | 86 | 85 | 83 | 82 |
| 2008 | 85 | 80 | 81 | 81 | 82 | 80 | 79 | 79 | 80 | 84 | 89 | 86 | 82 |
| 2009 | 82 | 78 | 81 | 82 | 83 | 74 | 78 | 78 | 79 | 83 | 83 | 85 | 80 |
| 2010 | 83 | 79 | 78 | 78 | 77 | 79 | 80 | 80 | 83 | 84 | 90 | 87 | 81 |
| 2011 | 87 | 80 | 85 | 81 | 81 | 79 | 80 | 80 | 81 | 86 | 86 | 86 | 83 |
| 2012 | 86 | 81 | 80 | 80 | 76 | 73 | 74 | 76 | 82 | 86 | 88 | 87 | 81 |
| 2013 | 85 | 84 | 81 | 82 | 84 | 81 | 82 | 81 | 80 | 86 | 88 | 86 | 83 |
| 2014 | 83 | 79 | 78 | 76 | 80 | 78 | 77 | 80 | 83 | 87 | 89 | 87 | 81 |
| 2015 | 84 | 80 | 79 | 80 | 79 | 81 | 78 | 83 | 84 | 87 | 91 | 88 | 83 |
| 2016 | 85 | 83 | 80 | 77 | 80 | 81 | 84 | 80 | 81 | 86 | 90 | 90 | 83 |

The data were retrieved from https://www.tmd.go.th, November 29, 2023
