# Supplementary material for: The enhancive effect of the 2014–2016 El Niño-induced drought on the control of soil-transmitted helminthiases without anthelmintics: A longitudinal study
Source: PLoS Negl Trop Dis. 2024 Jul 12;18(7):e0012331. doi: 10.1371/journal.pntd.0012331 (PMC11268648; doi:10.1371/journal.pntd.0012331)
Supplement: S6 Table — (DOCX) [file pntd.0012331.s006.docx]

**S6 Table. Prevalence and intensity of 3 soil-transmitted helminths by severity of infection, sex, and age in 2012.**

|  |  | **Prevalence and intensity**  **arithmetic**  **mean** |  |  |  |  |  |  |  |  |  |  |  |  |
| --- | --- | --- | --- | --- | --- | --- | --- | --- | --- | --- | --- | --- | --- | --- |
|  |  | ***Ascaris*** |  |  |  | ***Trichuris*** |  |  |  | **hookworm** |  |  |  |  |
|  |  | **Light infection** |  | **Moderate**  **infection** |  | **Light**  **infection** |  | **Moderate**  **infection** |  | **Light**  **infection** |  | **Moderate**  **infection** |  | **Heavy**  **infection** |
| **Age** | **n** | **Male** | **Female** | **Male** | **Female** | **Male** | **Female** | **Male** | **Female** | **Male** | **Female** | **Male** | **Female** | **female** |
| 5-10 | 17 | 0 | 0 | 11.8  8300 | 0 | 17.6  627 | 23.5  540 | 0 | 0 | 52.9  827 | 23.5  780 | 5.9  2000 | 5.9  2480 | 0 |
| 11-20 | 89 | 5.6  2196 | 3.4  3067 | 6.7  9967 | 9  7675 | 23.6  415 | 19.1  499 | 1.1  1040 | 2.2  1740 | 36  647 ^a^ | 40.4  875 ^a^ | 2.2  2300 | 0 | 1.1  6200 |
| 21-30 | 53 | 0 | 0 | 0 | 0 | 1.9  320 | 0 | 0 | 0 | 11.3  627 ^a^ | 17  464 ^a^ | 0 | 0 | 0 |
| 31-40 | 47 | 2.1  2000 | 4.2  2400 | 0 | 4.2  8900 | 2.1  300 | 6.4  347 | 0 | 0 | 17  463 | 38.3  542 | 0 | 0 | 0 |
| 41-50 | 48 | 2.1  1600 | 0 | 0 | 4.2  9500 | 14.6  320 | 22.9  341 | 0 | 2.1  8000 | 33.3  554 | 39.6  656 | 0 | 0 | 0 |
| 51-60 | 29 | 0 | 6.9  2200 | 0 | 6.9  8800 | 3.4  400 | 6.9  300 | 0 | 0 | 34.5  598 | 34.5  784 | 0 | 0 | 0 |
| 61-70 | 16 | 0 | 0 | 0 | 0 | 25  465 | 12.5  720 | 0 | 0 | 37.5  580 | 25  645 | 6.3  2400 | 0 | 0 |

^a^ P < 0.05
