# Supplementary material for: The enhancive effect of the 2014–2016 El Niño-induced drought on the control of soil-transmitted helminthiases without anthelmintics: A longitudinal study
Source: PLoS Negl Trop Dis. 2024 Jul 12;18(7):e0012331. doi: 10.1371/journal.pntd.0012331 (PMC11268648; doi:10.1371/journal.pntd.0012331)
Supplement: S7 Table — (DOCX) [file pntd.0012331.s007.docx]

| Rainfall in mm April 3-30, 2012  Rainfall in mm May 1-24, 2012  Rainy days  No rain (days) (0 mm)  Slight rain (days) (0.1-10 mm)  Moderate rain (days) (10.1-35 mm) | 0, 0, 0, 0, 0, 1.4, 1.6, 0.7, 0, 0, 0, 4.7, 1.6, 2.7, 0, 0, 0, 0, 0, 0, 0.2, 0.5, 0, 0, 0, 0, 19.1and 0  0, 0.2, 0, 0, 0, 2, 0, 9.5, 6.8, 0, 0, 0, 0, 0, 0, 0, 0, 0.6, 0, 0, 0, 0, 0 and 0  38  13  1 |
| --- | --- |

**S7 Table. Rainfall and rainy days after albendazole intake for 52 days from April 3 to May 24, 2012**

The data were retrieved from https://www.tmd.go.th
