# Supplementary material for: The enhancive effect of the 2014–2016 El Niño-induced drought on the control of soil-transmitted helminthiases without anthelmintics: A longitudinal study
Source: PLoS Negl Trop Dis. 2024 Jul 12;18(7):e0012331. doi: 10.1371/journal.pntd.0012331 (PMC11268648; doi:10.1371/journal.pntd.0012331)
Supplement: S8 Table — (DOCX) [file pntd.0012331.s008.docx]

**S8 Table.** **Prevalence and intensity (egg/gram of stool) of 4 soil-transmitted helminthiases in participants who received albendazole in 2012 only, living in village 11 and in surrounding areas from 2012-2016.**

| **Year** | **Prevalence and intensity of 4 STHs Arithmetic mean (SD) (range)** |  |  |  |  |
| --- | --- | --- | --- | --- | --- |
|  | ***A. lumbricoides*** | ***T. trichiura*** | **Hookworm** | ***S. stercoralis*** | **Any STHs** |
| *2008*  *N=296* | *19.6*  *4862.1(2643.3)*  *(1200-12000)* | *27.7*  *592.8 (313.0)*  *(100-1500)* | *68.6*  *770.7 (334.9)*  *(120-2200)* | *28.7*  *ND* | *80.4* |
| 2012  N=299 | 12.0  6255 (4108.9)  (780-18000) | 27.1  564.3 (888.1)  (60-8000) | 64.5  740.3 (561.2)  (120-6200) | 26.1  ND | 75.9 |
| 2013  N=299 | 5.7 ^b^  6663.5 (12138.7) ^a^  (780-46000) | 20.7  599.7 (2100.8)  (40-16800) | 39.8 ^a^  394.9 (198.2) ^a^  (40-1000) | 17.7 ^c^  ND | 56.9 ^a^ |
| 2016  N=282 | 1.8 ^b^  1640 (572.7) ^c^  (1000-2400) | 9.9 ^a^  139.3 (86)  (40-360) | 26.2 ^a^  204.7 (141.6) ^a^  (20-780) | 17.4  ND | 43.6 ^a^ |
| *2019*  *N=304* | *0.3*  *680 (0)*  *(680-680)* | *1.6*  *92 (48.2)*  *(40-160)* | *8.6*  *274.6 (361.2)*  *(40-1800)* | *8.9*  *ND* | *18.1* |
| *2023*  *N=225* | *0* | *0.4*  *80 (0)*  *(80-80)* | *4.4*  *202 (97.7)*  *(60-360)* | *4.9*  *ND* | *9.3* |

^a^ P < 0.001, ^b^ P < 0.01, ^c^ P < 0.05

ND, not done
