# Supplementary material for: The enhancive effect of the 2014–2016 El Niño-induced drought on the control of soil-transmitted helminthiases without anthelmintics: A longitudinal study
Source: PLoS Negl Trop Dis. 2024 Jul 12;18(7):e0012331. doi: 10.1371/journal.pntd.0012331 (PMC11268648; doi:10.1371/journal.pntd.0012331)
Supplement: S9 Table — (DOCX) [file pntd.0012331.s009.docx]

**S9 Table.** **Monthly rainfall and rainy days were measured by station 0747 in Ban Tha Ngam, one of the stations nearest to Village 11 of Mokhalan**.

|  | **Rainfall in mm (No. of rainy day)** | | | | | | | | | | | | |
| --- | --- | --- | --- | --- | --- | --- | --- | --- | --- | --- | --- | --- | --- |
|  | **Jan** | **Feb** | **Mar** | **Apr** | **May** | **Jun** | **Jul** | **Aug** | **Sep** | **Oct** | **Nov** | **Dec** | **Tot** |
| 2014 | ND | ND | ND | ND | 6.5  (1) | 2  (1) | ND | 0  (0) | 9  (3) | 0  (0) | 8.5  (3) | ND | 26  (8) |
| 2015 | 59  (9) | 2  (1) | 1  (1) | 63.5  (5) | 189  (12) | 79.5  (6) | 87  (10) | 259  (11) | 142  (8) | ND | ND | 28  (7) | 910  (70) |
| 2016 | 8  (1) | ND | ND | ND | ND | ND | 188.5  (10) | 49.5  (11) | 84  (11) | 140  (8) | 48  (9) | 43.5  (4) | 561.5  (54) |
| 2017 | 91  (6) | 179.5  (8) | 123  (7) | 234  (14) | 151.5  (14) | 125.5  (11) | 162.5  (9) | 104.5  (14) | 310.5  (18) | 168  (14) | 1253  (14) | 1093  (12) | 3996  (141) |
| 2018 | 245.5  (12) | 41.5  (5) | 50.5  (5) | 21  (4) | 187.5  (8) | 15.5  (8) | 31  (13) | 43  (16) | 181.5  (18) | 175  (19) | 299.5  (15) | 519  (13) | 1810.5  (136) |
| 2019 | 35  (9) | 0  (0) | ND | 0  (0) | 0  (0) | 0  (0) | 0  (0) | 0  (0) | 0  (0) | 0 (0) | ND | ND | 35  (9) |
| 2020 | ND | ND | ND | ND | ND | 0  (0) | 27  (2) | 0  (0) | 0.5  (1) | 19.5  (3) | 38.5  (7) | 6  (2) | 91.5  (15) |
| 2021 | 51  (4) | 10 (2) | 6  (1) | 123  (7) | 36  (6) | 20.5  (4) | 87  (11) | 168  (17) | 41  (5) | 3  (2) | 213  (6) | 253  (11) | 1011.5  (76) |
| 2022 | 88  (8) | 331.5  (12) | 132  (6) | 258  (10) | 92.5  (10) | ND | 13.5  (4) | 0.5  (1) | 0  (0) | 0  (0) | ND | ND | 916  (51) |
| 2023 | ND | 0.5  (1) | ND | 12  (1) | 77.5  (4) | ND | 19.5  (5) | 0  (0) | 65  (4) |  |  |  | 174.5  (15) |

The data were retrieved from https://www.thaiwater.net/weather/rain

ND, no data
