# Supplementary material for: The enhancive effect of the 2014–2016 El Niño-induced drought on the control of soil-transmitted helminthiases without anthelmintics: A longitudinal study
Source: PLoS Negl Trop Dis. 2024 Jul 12;18(7):e0012331. doi: 10.1371/journal.pntd.0012331 (PMC11268648; doi:10.1371/journal.pntd.0012331)
Supplement: S10 Table — (DOCX) [file pntd.0012331.s010.docx]

**S10 Table.** **Monthly rainfall and rainy days were measured by station 0064 Ban Nai Tub, one of the stations nearest to Village 11 of Mokhalan, and with the most complete data availability since 2014.**

|  | **Rainfall in mm (No. of rainy day)** | | | | | | | | | | | | |
| --- | --- | --- | --- | --- | --- | --- | --- | --- | --- | --- | --- | --- | --- |
|  | **Jan** | **Feb** | **Mar** | **Apr** | **May** | **Jun** | **Jul** | **Aug** | **Sep** | **Oct** | **Nov** | **Dec** | **Tot** |
| 2014 | ND | ND | ND | ND | 0  (0) | 4  (3) | 47  (4) | 7  (4) | 49.5  (3) | 0  (0) | 0  (0) | ND | 107.5  (14) |
| 2015 | 0  (0) | 1  (1) | 0  (0) | 133  (8) | 370  (10) | 116  (11) | 140  (9) | 124  (17) | 10  (8) | 1  (1) | 255  (4) | 156  (8) | 1306  (77) |
| 2016 | 0  (0) | 0  (0) | 0  (0) | 0  (0) | 0  (0) | ND | 217  (9) | 64.5  (16) | 122.5  (17) | 328  (17) | 257  (15) | 1190  (12) | 2179  (86) |
| 2017 | 1355  (17) | 150  (10) | 105  (8) | 369  (13) | 82  (14) | 113  (11) | 21  (15) | 42  (7) | 207  (15) | 134  (9) | 1149  (16) | 1030  (12) | 4757  (147) |
| 2018 | 117.5  (16) | 19  (5) | 17.5  (3) | 14  (6) | 3  (4) | 25.5  (23) | 50  (28) | 56  (20) | 29.5  (15) | 45.5  (26) | 571  (17) | 784  (15) | 1733  (178) |
| 2019 | 71  (4) | 9.5  (2) | ND | ND | 0  (0) | 5.5  (2) | 5  (1) | 4  (1) | 86  (4) | 14 (1) | ND | ND | 195  (15) |
| 2020 | ND | ND | ND | ND | ND | 35  (2) | 116  (6) | 47.5  (7) | 0.5  (1) | 0  (0) | 3.5  (4) | 14  (3) | 216.5  (23) |
| 2021 | 68  (3) | 0 (0) | 0  (0) | 0  (0) | 0  (0) | 0  (0) | 0  (0) | 0.5  (1) | 0  (0) | 0  (0) | 405.5  (14) | 196  (11) | 670  (29) |
| 2022 | 82  (7) | 606.5  (16) | 161  (6) | 7.5  (6) | 20.5  (13) | ND | ND | 27  (8) | 60.5  (4) | ND | ND | ND | 965  (60) |
| 2023 | ND | ND | ND | 4.5  (2) | 46.5  (3) | ND | 31.5  (5) | 15  (3) | 8.5  (3) |  |  |  | 106  (16) |

The data were retrieved from https://www.thaiwater.net/weather/rain

ND, no data
