# Supplementary material for: The enhancive effect of the 2014–2016 El Niño-induced drought on the control of soil-transmitted helminthiases without anthelmintics: A longitudinal study
Source: PLoS Negl Trop Dis. 2024 Jul 12;18(7):e0012331. doi: 10.1371/journal.pntd.0012331 (PMC11268648; doi:10.1371/journal.pntd.0012331)
Supplement: S11 Table — (DOCX) [file pntd.0012331.s011.docx]

**S11 Table.** **Prevalence and intensity (egg/gram of stool) of 4 soil-transmitted helminthiases in school-aged children (SAC) at Wat Mokhalan primary school who had received preventive chemotherapy (either a single dose of 400 mg albendazole or 500 mg mebendazole) since 2012.**

| **Year** | **Prevalence and intensity of 4 STHs Arithmetic mean (SD) (range)** |  |  |  |  |
| --- | --- | --- | --- | --- | --- |
|  | ***A. lumbricoides*** | ***T. trichiura*** | **Hookworm** | ***S. stercoralis*** | **Any STHs** |
| 2008  50% of SAC examined  (189/380) | 4.2  (8/189)  8350±3275.5  (4800-14000) | 19.1  (36/189)  782.8±257.5  (300-1800) | 50.8  (96/189)  910.3±373.1  (280-2600) | 13.2  (25/189)  ND | 58.7  (111/189) |
| 2013  41% of SAC examined  (78/188) | 3.9  (3/78)  5066.7±3900.4  (1200-9000) | 15.4  (12/78)  530±175.7  (240-820) | 42.3  (33/78)  840.6±295.4  (120-1800) | 10.3  (8/78)  ND | 44.9 ^a^  (35/78) |
| 2017  54% of SAC  examined  (75/140) | 0 | 5.3 ^a^  (4/75)  900±853.7  (280-2140) | 10.7 ^c^  (8/75)  217.5±84.5  (120-340) | 4.0  (3/75)  ND | 16 ^b^  (12/75) |

^a^ P < 0.05, ^b^ P < 0.001, ^c^ P < 0.00001

ND, not done
