# Supplementary material for: The enhancive effect of the 2014–2016 El Niño-induced drought on the control of soil-transmitted helminthiases without anthelmintics: A longitudinal study
Source: PLoS Negl Trop Dis. 2024 Jul 12;18(7):e0012331. doi: 10.1371/journal.pntd.0012331 (PMC11268648; doi:10.1371/journal.pntd.0012331)
Supplement: S12 Table — (DOCX) [file pntd.0012331.s012.docx]

**S12 Table. Response of STHs to albendazole 3 weeks after albendazole administration and to drought one year after long-lasting drought.**

|  | ***A. lumbricoides*** | ***T. trichiura*** | **Hookworm** | ***S. stercoralis*** |
| --- | --- | --- | --- | --- |
| **2012/2013** |  |  |  |  |
| No. of infected cases (2012) | 36 | 81 | 193 | 78 |
| No. of uncured ABZ cases | 0 | 45 | 39 | NA |
| No. of cured ABZ cases | 36 | 36 | 154 | NA |
| ABZ cure rate | 100 (36/36) | 44.4 (36/81) | 79.8 (154/193) | NA |
| Reinfected cases | 15 | 15 | 61 | NA |
| Reinfection rate | 41.7 (15/36) ^a^ | 41.7 (15/36) | 39.6 (61/154) ^b^ | NA |
| Newly infected cases | 2 | 2 | 19 | 4 |
| New infection rate | 0.76 (2/263) | 0.92 (2/218) ^c^ | 17.9 (19/106) ^a^ | 1.8 (4/221) |
| **2013/2016** |  |  |  |  |
| No. of infected cases (2013) | 17 | 62 | 119 | 53 |
| Reinfected cases | 3 | 19 | 6 | 2 |
| Reinfection rate | 8.3 (3/36) ^a^ | 52.8 (19/36) | 3.9 (6/154) ^b^ | NA |
| Newly infected cases | 2 | 9 | 1 | 3 |
| New infection rate | 0.76 (2/263) | 4.13 (9/218) ^c^ | 0.94 (1/106) ^a^ | 1.36 (3/221) |
| Naturally cured cases | 17 | 49 | 52 | 9 |
| Natural cure rate | 100 (17/17) | 79 (49/62) | 43.7 (52/119) | 17.6 (9/51) ^a*^ |
| No. of infected cases (2016) | 5 | 28 | 74 | 49 |

^a^ P <0.001 ^b^ P <0.00001 ^c^ P <0.05 ABZ, albendazole NA, not applicable

* Missing 2 patients
