# Supplementary material for: The enhancive effect of the 2014–2016 El Niño-induced drought on the control of soil-transmitted helminthiases without anthelmintics: A longitudinal study
Source: PLoS Negl Trop Dis. 2024 Jul 12;18(7):e0012331. doi: 10.1371/journal.pntd.0012331 (PMC11268648; doi:10.1371/journal.pntd.0012331)
Supplement: S13 Table — (DOCX) [file pntd.0012331.s013.docx]

**S13 Table. Comparative prevalence of trichuriasis between a control area where wet soil has been artificially maintained and in other areas with dry sandy soil of Village 4 Thasala Subdistrict located at the seashore 2013-2018.**

| Year | Prevalence and intensity of trichuriasis mean±SD (range) |  |  |
| --- | --- | --- | --- |
|  | Control area (n=20) | Other areas (n=108) | Pre-school children |
| 2013 | 100  1834±2357  (120-9600) | 35.2  677±375  (120-1600) | 35 (28/80)  779±533  (180-2420) |
| 400 mg albendazole administration | | | |
| 2014 | 85  1691±2247  (100-7500) | 28.7*  490±287  (60-1200) | ND |
| Drought occurred during Feb 2014- Mar 2015 | | | |
| 2016 | 90  1206±1786  (40-7000) | 9.3* (P=0.0003)  282±293  (80-840) | ND |
| 2018 | 65  1126±1410  (20-4000) | 6.5  269±279  (40-760) | 7.1 (2/28)  620±311  (400-840) |

The control area comprises three houses with roofs linked together to prevent sandy soil ground from sunlight. Three families who are relatives; 7 adults, and 13 children, have lived here. All were infected with *T. trichiura*. The habit of washing feet before entering the homes makes the soil wet all the time. Young children defecate on soil. This environment perfectly maintains the life cycle of *T. trichiura*.

Drinking water -tap water and commercially available drinking water.

Sanitation -all households have latrines. No sewage disposal.
