# Supplementary material for: The enhancive effect of the 2014–2016 El Niño-induced drought on the control of soil-transmitted helminthiases without anthelmintics: A longitudinal study
Source: PLoS Negl Trop Dis. 2024 Jul 12;18(7):e0012331. doi: 10.1371/journal.pntd.0012331 (PMC11268648; doi:10.1371/journal.pntd.0012331)
Supplement: S14 Table — (DOCX) [file pntd.0012331.s014.docx]

**S14 Table.** **Prevalence and intensity (egg/gram, larva/gram of stool) of 4 soil-transmitted helminths in people in isolated area 1 of village 11, 2008-2019.**

| Year | Prevalence and intensity of 4 soiled transmitted helminth infections (N=15)  mean±SD (Range) | | | | |
| --- | --- | --- | --- | --- | --- |
|  | *A. lumbricoides* | *T. trichiura* | Hookworm | *S. stercoralis* | Any STH infection |
| 2008 | 33.3  2160.0±952.9  (1200-3400) | 60.0  473.3±402.2  (100-1100) | 60.0  360±211  (120-800) | 46.7 | 93.3 |
| 2012 | 60.0  5133.3±3683.8  (1600-12000) | 33.3  580±323.7  (300-1040) | 80  695±537.8  (200-2200) | 46.7 | 100 |
| 400 mg albendazole administration for consecutive 3 days for *S. stercoralis* and a single dose for others | | | | | |
| 2013 | 33.3  18400±18535.9  (1400-46000) | 13.3  90±70.7  (40-140) | 73.3  534.6±359.0  (40-1000) | 20 | 80 |
| Drought occurred during Feb 2014- Mar 2015 | | | | | |
| 2016 | 0 | 6.7  120±0  (120) | 66.7  366±206.4  (160-780) | 40 | 86.7 |
| 2019 | 0 | 6.7  40±0  (40) | 26.7  860±709.7  (100-1800) | 26.7 | 53.3 |

Observed WASH status

Drinking water -unboiling groundwater

Sanitation -no latrine

Hygiene -47% walk barefoot when going outside
