# Supplementary material for: The enhancive effect of the 2014–2016 El Niño-induced drought on the control of soil-transmitted helminthiases without anthelmintics: A longitudinal study
Source: PLoS Negl Trop Dis. 2024 Jul 12;18(7):e0012331. doi: 10.1371/journal.pntd.0012331 (PMC11268648; doi:10.1371/journal.pntd.0012331)
Supplement: S15 Table — (DOCX) [file pntd.0012331.s015.docx]

**S15 Table. Prevalence and intensity (egg/gram, larva/gram of stool) of 2 soil-transmitted helminths in people in isolated area 2 of village 11, 2008-2019.**

| Year | Prevalence and intensity of 4 soiled transmitted helminth infections (N=13)  mean±SD (Range) | | | | |
| --- | --- | --- | --- | --- | --- |
|  | *A. lumbricoides* | *T. trichiura* | Hookworm | *S. stercoralis* | Any STH infection |
| 2008 | 0 | 0 | 100  852.3±351.6  (420-1500) | 53.9 | 100 |
| 2012 | 0 | 0 | 100  638.5±250.9  (240-1100) | 30.8 | 100 |
| 400 mg albendazole administration 3 consecutive days for *S. stercoralis* and a single dose for others | | | | | |
| 2013 | 0 | 0 | 76.9  ND | 0 | 76.9 |
| Drought occurred during Feb 2014- Mar 2015 | | | | | |
| 2016 | 0 | 0 | 38.5  ND | 0 | 38.5 |
| 2019 | 0 | 0 | 0 | 0 | 0 |

Observed WASH status.

Drinking water -unboiling groundwater or rainwater.

Sanitation -no latrine.

Hygiene -54% walk barefoot when going outside.
